# A full belly counsels well: More stabilisation with more responsibility

**DOI:** 10.1371/journal.pone.0344283

**Published:** 2026-03-06

**Authors:** Xiaoqi Sheng

**Affiliations:** School of Economics and Management, Southeast University, Nanjing, China; East China Normal University, CHINA

## Abstract

**Design/methodology/approach:**

This paper uses the data of Chinese A-share listed companies from 2009 to 2022 to study the impact of supply chain stability on corporate ESG performance.

**Purpose:**

To study the impact and mechanism of supply chain stability on corporate ESG performance.

**Findings:**

This paper found that the improvement of supply chain stability can improve the ESG performance of firms, and this conclusion still holds after the instrumental variables method, systematic GMM method, PSM method, omitted variables test and Double Machine Learning (DML) approach, and the improvement of supply chain stability can optimise the ESG performance of firms through the channels of reducing the corporate risk-taking, reducing the agency costs, and reducing the financing constraints, and this facilitating effect is more significant in the large firms, firms with higher-standard audit supervision, firms located in western regions, and non-technology-intensive firms.

**Practical implications:**

The findings of this paper can provide a realistic framework for national and local governments to actively promote the stable development of supply chains in order to achieve sustainable economic development.

**Social implications:**

This paper deepens the understanding of the external stakeholders of enterprise sustainable development, and provides an opportunity to actively play the role of external stakeholders in monitoring the development of enterprises, participate in corporate governance of enterprises, and achieve a win-win situation of supply chain stability and environmental sustainability.

**Originality/value:**

This study contributes to the literature by shedding light on the relationship between supply chain stability and ESG in the context of external stakeholders.

## 1. Introduction

Climate change is increasingly affecting food security, the poverty reduction process, ecosystems and other key areas, as emphasized in the Global State of the Climate Report 2023 (WMO). It is projected that global temperatures will rise by 1.45°C by 2023, highlighting the urgency of reducing emissions to alleviate extreme weather, sea level rise and ecosystem degradation [1]. As governments and financial markets around the world deepen their understanding of the significant risks brought about by climate change, enterprises are facing increasing pressure to proactively respond. Effective governance mechanisms and responsible industrial practices are crucial for reducing environmental damage and complying with emerging regulations including carbon pricing [2,3]. Against this backdrop, ESG ratings, as an important indicator for measuring the sustainable development performance of enterprises, are increasingly influencing investment decisions and public perception [4,5]. The high attention paid to ESG elements indicates that enterprises not only need to take comprehensive environmental responsibility measures to reduce risks, but also can enhance market competitiveness and long-term development resilience. As the status of ESG in enterprise management and policy-making continues to rise, enhancing enterprises’ understanding and management capabilities of climate risks has become a core topic in academic research and policy discussions.

Since the UN Principles for Responsible Investment was proposed in 2006, the importance of environmental, social and governance (ESG) factors in supply chain management has become increasingly prominent. The key subjects promoting ESG practices in the enterprise supply chain include suppliers and consumers, who play a core role in formulating and implementing ESG policies [6]. In recent years, disturbances such as the COVID-19 pandemic and geopolitical crises have severely affected the reliability of the global supply network, leading to supply chain decoupling and significant economic impacts [7,8]. In countries such as Turkey and India, these disturbances have even had a significant impact on the stock market [9]. The operational performance and innovation ability of enterprises largely depend on the stability of the supply chain, including the interaction with suppliers and customers [10]. A stable supply chain can enhance an enterprise’s ESG performance by optimizing internal resource allocation, reducing transaction costs, and improving resource availability [11]. However, previous studies have mainly focused on the financial consequences of supply chain stability, such as credit risk and financial distress, while paying less attention to its relationship with ESG performance [12]. Given the increasingly prominent global issue of sustainable development, this gap in the existing literature highlights the necessity of in-depth research on how supply chain stability affects the ESG performance of enterprises.

The distribution and stability of enterprise suppliers and customers play a key role in determining the stability of the supply chain [13]. The degree of dependence of enterprises on upstream and downstream stakeholders significantly affects their strategic decisions [14]. Existing studies have shown that by enhancing the ability of resource integration and reducing production downtime, supply chain concentration at both the supplier and customer levels can positively affect the ESG performance of enterprises [15]. In the context of sustainable development, reliable supply chain relationships help enhance corporate responsibility and promote more efficient resource allocation [16]. Conversely, a decentralized supply chain characterized by diverse suppliers and customers can reduce risks and thereby further improve ESG performance [17]. Although highly centralized supply chains may weaken an enterprise’s bargaining power, decentralized supply chains provide greater flexibility for enterprises, enabling them to adapt to changes and drive innovation [18]. Research also shows that enterprises with a higher degree of supply chain diversification are more capable of strengthening governance mechanisms and improving ESG performance [19]. Based on this, this paper aims to explore how supply chain stabilityaffects the ESG performance of enterprises, thereby enriching the research literature on the impact of external factors on ESG practices.

This research mainly makes contributions in three aspects. Firstly, this study expands the intersection of supply chain research and ESG research, correlating the ESG performance of enterprises with the stability of the supply chain. The existing literature mainly focuses on the impact of supply chain stability on financial outcomes such as stock price fluctuations, financing costs, and risk-taking [20,21], while the research on how it affects the ESG performance of enterprises is still very limited. This article emphasizes the significant role of external stakeholders, especially supply chain partners, in the sustainable development of enterprises, making up for the deficiencies of existing research in this regard. Secondly, this study constructs an integrated theoretical framework based on the resource dependence theory and the social capital theory to explain how supply chain stability affects enterprises’ investment in the environment, society and governance. Compared with existing studies that mainly focus on the internal governance mechanisms of enterprises or ESG financial incentives [22,23], this study emphasizes that stable supply chain relationships can enhance resource availability, promote information flow and strengthen relationship trust, thereby deepening the understanding of how external structural factors shape the ESG performance of enterprises. Finally, this paper provides systematic empirical evidence that supply chain stability significantly enhances the ESG performance of enterprises, and its impact is more pronounced in large enterprises, highly audited and regulated enterprises, enterprises in the western region, and non-technology-intensive enterprises. This conclusion differs from the perspectives of existing studies that mainly focus on the eastern region or technology-intensive enterprises [24,25], further indicating that the government can effectively promote the sustainable development of enterprises through market-oriented mechanisms.

## 2. Literature review and theoretical hypotheses

### 2.1. Literature review

#### 2.1.1. Impact of supply chain stability on business operations.

Supply chain management is a crucial aspect of corporate management [26]. A robust upstream and downstream supply chain is a formidable catalyst for firms to advance sustainable development against intense market competition [27]. Supply chain stability indicates the enterprise’s capacity to negotiate and maintain strong relationships with both upstream and downstream partners [28]. The stability of the supply chain is expressed by the stability of both suppliers and customers.

Supplier stability refers to the level of fragmentation between a company and its upstream suppliers, which also indicates its operational efficiency to some extent [29]. Establishing secure supplier connections can decrease the expenses related to uncertain contracts, alleviate challenges in production budgeting and inventory turnover, and improve the financial stability of the company [30]. Nevertheless, long-term supplier stability may lead to over-reliance on certain suppliers, potentially reducing firms’ bargaining power and limiting access to alternative resources in times of crisis [31]. Although the firm may have a somewhat adverse bargaining strength, having stable and reliable suppliers can effectively minimise the enterprise’s selling expenses, according to the main and quantity profit analysis [32]. Long-term contracts create economies of scale in the supply of goods and resources, resulting in lower unit costs and avoiding the need for additional time and inefficient information search owing to transaction uncertainty ([33]. Moreover, enhanced supplier stability can foster the development of a cohesive community of interest between enterprises and suppliers [34]. This facilitates more effective resource integration, information sharing, and technological advancements for enterprises. Additionally, it enables the formation of a shared interest through a stable and strategic cooperative relationship. Under the supply chain perspective, market competition shifts from being solely between individual suppliers to being between distinct supply networks [35]. Due to the strong interdependence between organisations and suppliers, leveraging the supply chain for channel integration and resource integration becomes a more feasible approach to develop a competitive strategic advantage [36]. However, this perspective assumes a static competitive environment, while in dynamic markets, excessive reliance on supplier stability may reduce firms’ agility and responsiveness to innovation [35].

Customer stability refers to the level of dispersion between a company and its customers, which indicates the stability of the company’s competitiveness and customer loyalty [37]. Variations in customer loyalty can indicate disparities in market competitiveness among firms and their capacity to negotiate with customers. Establishing a long-term and stable cooperative relationship between the firm and its customers might contribute to higher customer stability [38]. This contributes to enhancing the efficacy of product turnover, diminishing the cost of goods sold and administrative expenses of the enterprise, hence augmenting the income of the enterprise. However, excessive customer concentration may expose firms to significant revenue risks if key customers shift to competitors [39]. Higher client concentration implies a limited number of customers. In this scenario, organisations can gain a deeper understanding of the preferences of their primary clients, improve their business processes, and successfully meet the demand for certain products [40]. This not only fosters the development of robust collaborative partnerships, but also diminishes the need for customer-related workforce and the expenses associated with searching for information. Simultaneously, prominent clients, as crucial stakeholders and intangible assets of the organisation, exert a substantial influence on the organization’s business decisions and financial strategies [41]. Furthermore, the interrelation between prominent clients and the organisation will also limit the organization’s options [42]. This suggests a potential trade-off between customer stability and strategic flexibility, an aspect that has received limited empirical exploration [41].

#### 2.1.2. Factors affecting corporate ESG performance.

The ESG performance of an enterprise is a metric that evaluates the sustainability of an enterprise by analysing its environmental, social, and governance elements. The current body of research primarily examines the primary elements that influence business ESG performance at the macro, meso, and micro levels.

Government policies at the macro level significantly influence corporate ESG disclosure, and the variation in the quality of corporate ESG disclosure across different cities can partially indicate the level of importance local governments place on corporate ESG development. The absence of consistency in disclosure rules is a significant factor contributing to the limited comparability of corporate ESG information. While stricter disclosure regulations may enhance transparency, they may also impose additional compliance costs on firms, potentially discouraging voluntary ESG initiatives [43]. The essence of the ESG idea is closely connected to the worldwide environmental movement and regulations concerning the environment. In their study, some scholars examined the yearly corporate reports and environmental reports of six industries: chemical, oil, gas, energy, automobile, and casualty insurance [44]. They discovered that countries and regions that have ratified the Kyoto Protocol tend to have higher levels of pollution and greenhouse gas (GHG) disclosures, as well as more stringent environmental regulations. Companies in countries with stronger environmental commitments and regions with well-defined carbon emissions trading guidelines tend to provide more comprehensive ESG disclosures [45]. However, the effectiveness of these policies in driving substantive corporate sustainability actions, rather than mere compliance, remains debated [46].There is evidence indicating a positive relationship between carbon disclosure requirements imposed by governments and stock exchanges and the governance of firms’ environmental disclosure quality [47]. Additionally, government environmental subsidies play a mediating role in this mechanism, suggesting that firms’ ESG behaviour can assist them in obtaining higher carbon emission allowances [46]. China has not implemented legislation for ESG reporting, as there are less strict audit requirements for multinational organisations. This is in contrast to most other governments that have established clear criteria. The absence of well-defined disclosure rules for ESG-related disclosures among Chinese enterprises has resulted in a higher level of autonomy in their information disclosure practices. Consequently, there has been a rise in the dissemination of insufficient data and intentional downplaying of facts. This obviously poses a challenge for third-party rating agencies to conduct a comprehensive comparison of Chinese companies’ ESG performance within the industry, hence hindering their ability to provide an unbiased and precise evaluation [48]. The presence of subjectivity in the environmental disclosure of Chinese enterprises creates a gap between the relevance, trustworthiness, comparability, and consistency of their ESG reporting information and that of other countries worldwide. The presence of significant geographical variation in the extent to which Chinese enterprises disclose information about the environment is worth mentioning. The proportion of firms that disclose information in Chinese provinces such as Jiangsu, Zhejiang, and Guangdong is less than 30%. In contrast, the disclosure rate in Beijing and Shanghai is as high as 40%, and even reaches 50% in Yunnan Province [49]. This phenomena not only signifies the impact of regional economic variables, but also indicates, to some degree, the variations in the significance that local governments place on environmental regulations.

At the meso level, the level of market competition has an impact on the ESG performance of companies. Additionally, external stakeholders, such as the media, have a significant influence on the information disclosure of companies through public opinion. Institutional investors, analysts, and rating agencies, with their professional teams, also influence companies’ inclination to disclose ESG information due to regulatory pressures, while simultaneously promoting the sound and steady growth of the capital market. Yet, excessive pressure from institutional investors may also incentivize greenwashing, where firms manipulate ESG disclosures to enhance their reputations without making substantive sustainability improvements [50]. Based on empirical examination of firm data from 60 nations or regions, it can be inferred that in a market with a larger size or higher industry substitutability, enterprises will experience more intense market rivalry, resulting in a reduced inclination to reveal information [51]. This is due to corporations’ desire to prevent competitors from acquiring supplementary information. Non-governmental organisations (NGOs) and the general public are significant external stakeholders that have an impact on a company’s ESG performance [52]. The media willingly dedicates time and resources to analyse and report on firms that demonstrate commendable ESG performance, as well as contribute to the betterment of society [53]. This increased media coverage enables these companies to attract greater attention from investors. Companies also utilise ESG as a strategic public relations instrument to cultivate a favourable societal perception and convey an optimistic message to the market. In order to satisfy the need for sustainable investment, institutional investors will not only consider the financial performance of companies but also integrate ESG disclosure into the analysis framework when making investment choices [25]. Moreover, an increased proportion of institutional investors’ ownership can efficiently control opportunistic behaviours resulting from the concentration of shareholdings, encourage greater transparency in management’s information sharing, and establish a positive cycle of corporate ESG development [54].

At the micro level, the composition of a company’s shareholders, the kind of ownership, the size of the company, and the qualities of its executives all have an impact on the company’s ESG performance, as well as its level of openness in providing information. An individual provides evidence that the consolidation of ownership and management inside businesses, as well as the national ownership, negatively affects the quality of companies’ reports [55]. According to data from JPMorgan Chase, a significant number of prominent companies are actively sharing information on their ESG practices. In 2022, over 80 per cent of the companies listed in China’s CSI 300 index had published ESG reports [56]. According to stakeholder theory, firms prioritise not only their financial success but also the achievement of societal value. Big corporations engage a greater number of stakeholders in their activities, which consequently exposes them to a higher level of external pressures. As a result, companies are motivated to improve their ESG performance to meet the expectations of their stakeholders and preserve a positive public perception. Simultaneously, corporate management may also be motivated to improve investor relations management by disclosing ESG information [57]. This can help mitigate the potential adverse effects of unfavourable information on investor sentiment. Nonetheless, some scholars suggest that internal ESG governance structures remain underdeveloped in many firms, leading to inconsistencies between disclosed ESG goals and actual business practices [58]. Management can overstate the ESG performance of a company to manipulate share prices and optimise resource allocation, so disseminating too positive information to the market. If the actual operational circumstances of a company are exposed, there is a possibility that the share price could significantly decline [59]. Moreover, the act of disclosing ESG reports can lead to a substantial rise in the operational expenses of companies. When the specific information preferences of stakeholders are not known, companies often opt to stay quiet until they have determined the preferences of the information users. Only then do they make tailored disclosures to appease them [60].

Although existing research has extensively explored the factors influencing corporate ESG performance from macro, meso and micro levels, the focus on how supply chain stability, a key external structural factor, shapes corporate ESG performance remains rather limited. Most of the existing literature focuses on the impact of upstream and downstream stability on the economic or operational results of enterprises, while its role in the sustainable development performance of enterprises and its internal mechanism still lack systematic research. Furthermore, current research rarely integrates and explains from the perspectives of resource dependence and social capital how supply chain stability affects a company’s ESG investment. To make up for the above deficiencies, this paper systematically examines the impact of supply chain stability on the ESG performance of enterprises and further reveals the internal mechanisms by which it functions by reducing enterprise risk-taking, lowering agency costs and alleviating financing constraints.

## 3. Theoretical hypotheses

According to the Resource Dependence Theory (RDT) and the Social Capital Theory (SCT), the stability of supply chain relationships plays a significant role in enhancing the ESG performance of enterprises [61,62]. From the perspective of RDT, maintaining long-term and stable cooperative relationships between enterprises and key suppliers and customers helps ensure the continuous and reliable supply of key resources such as raw materials, technology, information and funds, reduces uncertainties in the business process and enhances financial stability [63,64]. When resource acquisition is more predictable, enterprises can carry out long-term environmental protection investment, social responsibility projects or governance improvements with greater security, such as upgrading energy-saving equipment, promoting social public welfare activities and optimizing employee benefits, etc. [65]. From the perspective of SCT, stable supply chain relationships contribute to the accumulation of cross-enterprise social capital, including trust, reputation and the formation of cooperative norms [66,67]. This kind of social capital can promote knowledge sharing, technological collaboration and risk sharing, such as jointly conducting green technology research and development or collaboratively formulating environmental standards, thereby enhancing the overall environmental governance capacity and social responsibility performance of enterprises [68]. In addition, stable supply chain cooperation can enhance information transparency and division of responsibilities, reduce opportunistic behaviors such as information manipulation or evasion of environmental responsibilities by management, and thereby improve the quality of internal governance [69]. It can be seen that the stability of the supply chain not only enhances an enterprise’s resource guarantee capacity and governance efficiency, but also increases the trust of stakeholders such as investors, suppliers and customers in the enterprise, thereby prompting the enterprise to take more proactive sustainable actions in environmental, social and governance aspects. This positive cycle helps enterprises build a good reputation and attract more partners to jointly promote ESG practices. Based on the above theoretical analysis, this study proposes the following hypotheses:

**H1.** Enhancing supply chain stability will enhance the ESG performance of firms.

A stable supply chain relationship can indirectly promote ESG practices by reducing the risk-taking of enterprises. According to the Resource Dependence Theory (RDT), stable access to key resources and business credit helps alleviate the financial risks and operational uncertainties faced by enterprises [70]. Enterprises can obtain predictable raw material supplies, capital flow support and business credit in a long-term and stable supply chain network. This not only reduces the operational risks caused by supply disruptions or tight cash flow, but also reduces the reliance on high-cost emergency procurement or financing [71]. Furthermore, a stable supply chain relationship can alleviate the market risks faced by enterprises, such as price fluctuations, contract breaches or changes in customer demands, thereby reducing the uncertainties of enterprises in investment and operational decisions [72]. From the perspective of social capital Theory (SCT), long-term and stable cooperative relationships within the supply chain contribute to the formation of trust and cooperative norms, and reduce the risks brought about by opportunistic behaviors and information asymmetry [68]. Sharing market information, jointly formulating environmental protection standards or jointly carrying out social responsibility projects between suppliers and customers can all reduce transaction costs and strategic execution risks [73]. In a low-risk environment, enterprises can more effectively invest resources in ESG projects such as environmental protection, social responsibility, and governance improvement without overly worrying about short-term financial pressure or sudden operational problems [74]. In addition, stabilizing the supply chain also has a cross-enterprise transmission effect. When a certain enterprise in the supply chain actively promotes ESG practices, these standards can spread to upstream and downstream partners through a long-term cooperative network, forming a virtuous cycle within the supply chain [75]. On the one hand, when suppliers adopt environmental protection measures in the process of fulfilling contracts and cooperation, it can promote client enterprises to simultaneously enhance their ESG practice levels, thereby driving the sustainable development of the entire supply chain [76]. On the other hand, the concern of customers, especially large enterprises or end consumers, for social and environmental responsibility will also exert pressure on supply chain enterprises, prompting them to adopt more responsible practices to maintain market access and customer loyalty [77]. Therefore, a stable supply chain not only directly improves the operational environment of enterprises by reducing their risk-taking, but also indirectly promotes the enhancement of ESG performance through network and customer pressure.

A stable supply chain relationship can also enhance ESG performance by reducing agency costs. According to agency theory, agency problems often exist within enterprises and in supply chain networks, that is, management may deviate from the long-term goals of shareholders and other stakeholders for the maximization of its own interests [78]. A stable supply chain relationship helps alleviate this problem by strengthening the interdependence and information flow among enterprises. Long-term and stable cooperative relationships enable suppliers and customers to obtain enterprise operation information in a timely manner, reduce information asymmetry and opportunistic behavior, and thus make it more difficult for management to make short-sighted or self-interested decisions [79]. Furthermore, stable cooperative relationships reduce operational disruptions and high-cost adjustments for enterprises caused by the bankruptcy of partners or contract breaches, further lowering agency risks [80]. External supervision mechanisms also play a significant role in this, including the oversight of institutional investors, regulatory authorities and the media. These forces can impose constraints on the management of enterprises, promoting them to adhere to transparent and responsible governance principles and implement effective ESG measures [65]. Enterprises with lower agency costs can allocate resources more effectively to long-term environmental and social responsibility goals, such as investing in carbon emission management, employee benefits or social welfare projects [81]. Overall, supply chain stability enables enterprises to take more proactive actions in environmental, social and governance aspects by enhancing information transparency, reducing opportunistic behavior by management, strengthening internal governance and external supervision, thereby improving overall ESG performance.

Finally, a stable supply chain relationship can enhance an enterprise’s financing capacity, reduce financing constraints, and thereby support ESG investment. According to the Resource Dependence Theory (RDT), a long-term stable supply chain relationship can ensure the predictable flow of key resources and business credit of enterprises. This not only reduces financial uncertainty in operations but also enhances the ability of enterprises to obtain external funds [81]. When enterprises maintain stable cooperative relationships with core suppliers and customers, financial institutions and investors are more inclined to trust the enterprises’ debt-paying ability and operational stability, thereby providing lower-cost financing or more flexible loan terms [82]. From the perspective of social capital theory (SCT), the accumulation of trust and reputation in the supply chain helps promote the efficient flow of capital among network members [66]. Stable upstream and downstream relationships can enhance an enterprise’s credit rating within the supply chain, enabling it to obtain low-cost internal or external financing to support long-term sustainable projects, such as environmental protection measures or social responsibility activities [83]. In addition, supply chain stability has also improved information transparency and governance mechanisms, enabling financial institutions to assess enterprise risks more accurately, reduce financing costs caused by information asymmetry, and supervise the effective use of funds at the same time [84]. By reducing financing constraints, enterprises can invest resources more flexibly in ESG-related activities, such as purchasing energy-saving equipment, investing in green technologies, improving employee benefits or launching community public welfare projects [85]. This not only alleviates the constraints of short-term financial pressure on long-term ESG strategies, but also helps enterprises form a stable and sustainable development cycle.

**Fig 1:**
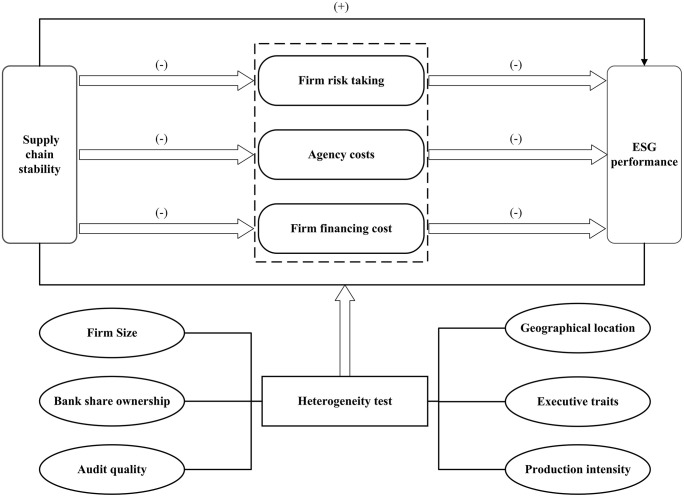
Research framework.

Further, this article suggests three hypothesis on how enhanced supply chain stability impacts firms’ ESG performance and Fig 1 depicts the research framework underpinning this study:

H2. Increased supply chain stability improves ESG performance by reducing firm risk taking.

H3. Increased supply chain stability improves ESG performance by reducing agency costs.

H4. Increased supply chain stability improves ESG performance by reducing firm financing cost.

## 4. Methodology

### 4.1. Sample selection and data sources

This study focuses on analysing the effects, mechanisms, and economic outcomes of supply chain stability on the ESG performance of Chinese A-share listed businesses. The data from 2009 to 2022 is used as the research sample. During this period, ESG practices gained prominence, particularly in the late 2010s, as policy and market demands intensified. The 2009–2022 period also aligns with the attention of international trade and supply chain stability, providing relevant data for the study. Overall, this timeframe allows us to capture significant changes in ESG practices and supply chain dynamics, reflecting the impact of external pressures and policy shifts. The original data is processed using the following criteria: (1) research samples with industry classifications of finance, insurance, ST, and ST* are excluded, (2) research samples with significant missing data are excluded, and (3) to mitigate the influence of outliers on the experimental results, all selected continuous variables undergo a 1% two-sided Winsorize shrinkage, (4) companies that do not disclose the specific names and specific sales of their top five customers are excluded, while companies that do not disclose information about their top five customers for two consecutive years are excluded for the purpose of calculating customer stability.This process results in a final set of 3530 firm-year observations. The ESG ratings are sourced from the CSI ESG ratings in the Wind database, while the supply chain data and firm financial data are obtained from the CSMAR database and the Wind database.

### 4.2. Definition of variables

#### 4.2.1. Dependent variable.

ESG rating (ESG_grade) is the core explanatory variable of this paper. This article utilizes the CSI ESG rating to assess the environmental, social, and governance (ESG) performance of organizations. The CSI ESG rating system is developed based on a standardized and internationally aligned ESG framework and integrates widely accepted evaluation methodologies with extensive practical experience. The rating standard encompasses 16 themes across three dimensions: environment, society, and corporate governance. These themes include climate change, resource utilization, environmental pollution, human capital, social contribution, data security and privacy, credit quality, governance risk, external sanctions, and business ethics, among others. In this study, the CSI ESG outcomes are converted into a 1–9 scale, where a higher score indicates stronger ESG performance and a lower score represents weaker performance.

#### 4.2.2. Independent variable.

The core explanatory variable of this paper is supply chain stability, including two indicators of supplier stability (SuppSt) and customer stability (Cust_stale). Starting in 2012, the China Securities Regulatory Commission (CSRC) has released an updated guideline for annual reports of listed companies. This guideline emphasizes that companies should disclose the names of their top 5 suppliers and the amount of goods they procure, as well as the names of their top 5 customers and their sales figures. This additional information enhances the availability of data for research on supply chain stability. This study quantifies supplier stability by computing the procurement ratio of the top five suppliers. Similarly, customer stability is assessed by calculating the ratio of stable customer sales to the sales of the top five customers. An enterprise’s business becomes increasingly reliant on reliable suppliers and customers as its supply chain stability increases.

### 4.3. Model settings

To investigate the relationship between supply chain stability and corporate ESG performance, this paper constructs the following econometric model:


$$ES{G\_grade}_{it}=\beta_{\text{0}}+\beta_{\text{1}}{Chain\_Stability}_{it}+\beta_{\text{2}}Controls_{it}+{Year}_{t}+Industry+\epsilon_{it}$$ (1)

Where $ES{G\_grade}_{it}$ denotes the ESG rating of the enterprise, ${Chain\_Stability}_{it}$ denotes the supply chain stability of the enterprise, which will be measured in different ways in this paper in order to verify the robustness of the regression results, $Controls_{it}$ are the various control variables, the specific names and definitions of the variables are shown in Table 1, i is used to distinguish between different listed companies, t is used to distinguish between different annual units, $\epsilon_{it}$ is the random disturbance term, and β is used to indicate the regression coefficient of each explanatory and control variables. In addition, the model controls for industry fixed effects (Industry) and time fixed effects (Year) and adjusts for standard errors clustering at the firm level.

**Table 1:** Variables and definitions.

Variables	Definition
Panel A	Dependent variable
ESG rating (ESG_grade)	According to CSC ESG rating from low to high assigned values of 1–9
Panel B	Independent variable
Supplier stability (SuppSt)	A measure of the proportion of purchases made by a company’s top five suppliers
Customer stability (Cust_stale)	Stable customer sales accounted for the top five customer sales proportion
Panel C	Control variables
Accounts receivable ratio (REC)	Net accounts receivable/total assets
Deposit ratio (INV)	Net inventory/total assets
Quick ratio (Quick)	(Current assets – inventory)/ current liabilities
Turnover of total assets (ATO)	Total sales revenue/Average total assets
Proportion of fixed assets (FIXED)	Net fixed assets/total assets

## 5. Empirical results

### 5.1. Baseline results

The benchmark regression results are displayed in Table 2. Columns (1)-(2) illustrate the influence of supplier stability and customer stability on firms’ ESG performance, respectively. The findings indicate that enhancing firms’ supply chain stability can lead to an improvement in their ESG performance. Moreover, the regression coefficients are significantly positive at the 1% significance level. (3)-(4) After adjusting for the control variables, the regression analysis confirms a significant positive relationship between supply chain stability and ESG performance. This suggests that higher levels of supplier stability and customer stability are associated with better ESG performance in the enterprise, thus supporting H1. The regression analysis consistently demonstrates that the coefficient of supplier stability is greater than the coefficient of customer stability across all four columns of the findings. This indicates that supplier stability has a more substantial influence on corporate ESG.

**Table 2:** Baseline results.

	(1)	(2)	(3)	(4)
	ESG_grade	ESG_grade	ESG_grade	ESG_grade
SuppSt	0.2505***		0.2451***	
	(0.0874)		(0.0871)	
Cust_stale		0.1938***		0.1834***
		(0.0671)		(0.0684)
Constant	3.7788***	3.7917***	3.9330***	3.9584***
	(0.0499)	(0.0480)	(0.1085)	(0.1071)
*Control*	Yes	Yes	Yes	Yes
*Industry FE*	Yes	Yes	Yes	Yes
*Year FE*	Yes	Yes	Yes	Yes
*N*	3530	3530	3530	3530
*R* ^*2*^	0.0856	0.0850	0.0909	0.0902

Note: The report includes robust standard errors that are clustered at the firm level, indicated in brackets. ***p < 0.01; **p < 0.05; *p < 0.1.

### 5.2. Endogenous test

#### 5.2.1. Instrumental variable estimation.

We employ the third power of the discrepancy between supplier stability and the average supplier stability in the corresponding industry (referred to as Lewbel IV1), as well as the third power of the discrepancy between customer stability and the average customer stability in the same industry (known as Lewbel IV2), as instrumental variables [86]. These instrumental variables are constructed using the higher order moments approach. Afterwards, we employed the 2SLS approach to ascertain the causal connection between the stability of enterprises’ supply chains and their ESG scores. The findings of the regression analysis are displayed in Table 3. The first-stage regression coefficients of IV in columns (1) and (2) show a strong positive correlation at the 1% level with both SuppSt and Cust_stale. In the second-stage regression, the coefficients of SuppSt and Cust_stale are statistically significant and positively correlated, indicating that enterprises with a stable supply chain can experience a considerable improvement in their ESG performance. The central finding remains valid even after accounting for the endogeneity problem.

**Table 3:** Instrumental variables method.

	First-stage		Second-stage	
	(1)	(2)	(3)	(4)
	SuppSt	Cust_stale	ESG_grade	ESG_grade
SuppSt			0.2363**	
			(0.0994)	
Cust_stale				0.2457***
				(0.0732)
Lewbel IV1	4.3866***			
	(0.1320)			
Lewbel IV2		4.1192***		
		(0.0898)		
*Control*	Yes	Yes	Yes	Yes
*IndustryFE*	Yes	Yes	Yes	Yes
*Year FE*	Yes	Yes	Yes	Yes
*Kleibergen-Paap rk LM*			277.9827	301.8172
*Kleibergen-Paap rk Wald F*			1104.6668	2102.6461
*N*	3530	3530	3530	3530

Note: The Cragg-Donald Wald F-statistics reject the original hypothesis at the 1% significance level, and the Kleibergen-Paap rk Wald F-statistics have values exceeding the 10% critical value (16.38), indicating that the choice of instrumental variables passes both the under-identification test and the weak identification test. The firm-level clustering is accounted for, and the corresponding robust standard errors are presented in brackets. ***p < 0.01; **p < 0.05; *p < 0.1.

#### 5.2.2. System GMM method.

In order to thoroughly evaluate the potential issue of endogeneity resulting from omitted variables and reverse causation, we employ the system generalised method of moments (GMM) to estimate our primary findings. The regression results are presented in Table 4. Our analysis reveals that the coefficients of SuppSt and Cust_stale are statistically significant at the 5% level. This indicates that the stability of enterprises’ supply chains has a favourable influence on their ESG performance. These findings further validate the reliability of our results.

**Table 4:** System GMM method.

	(1)	(2)
	ESG_grade	ESG_grade
L.ESG_grade	0.4819***	0.4245***
	(0.0800)	(0.0874)
SuppSt	0.7240**	
	(0.3421)	
Cust_stale		0.8279**
		(0.3228)
Constant	1.8372***	1.9302***
	(0.3463)	(0.4236)
*Control*	Yes	Yes
*Industry FE*	Yes	Yes
*Year FE*	Yes	Yes
*N*	2419	2419
*AR(1) P-value*	0.0000	0.0000
*AR(2) P-value*	0.2090	0.5799
*Hansen P-value*	0.2992	0.1546

Note: The P-value for AR(1) is less than 0.1, the P-value for AR(2) is greater than 0.1, and the P-value for Hansen is also greater than 0.1. These diagnostic tests collectively confirm the validity of the two-step system GMM dynamic model. The report includes the robust standard errors enclosed in brackets. ***p < 0.01; **p < 0.05; *p < 0.1.

#### 5.2.3. Propensity score matching (PSM).

The stability of a firm’s supply chain relationship is not random, which suggests the presence of a self-selection problem. To address this issue, we employ the propensity score matching method (PSM) to alleviate the problem of endogeneity. We categorise firms’ supply chain stability into treatment and control groups based on the median of the sample. If a firm’s supplier/customer stability is above the median, it is classified as a treatment group. Conversely, if it is below the median, it is classified as a control group. Subsequently, we utilised a logit model to pair the samples with their k-Nearest neighbors matching (k = 4), and the covariates employed in the matching procedure were in line with the control variables utilised in the baseline regression. The outcomes of the correlated regressions are presented in Table 5. After addressing the issue of self-selection bias, the study reveals that the coefficients of SuppSt and Cust_stale are positively significant at the 5% level. Our conclusions remain strong and reliable.

**Table 5:** PSM method.

	(1)	(2)
	ESG_grade	ESG_grade
SuppSt	0.2265**	
	(0.0888)	
Cust_stale		0.1758**
		(0.0692)
Constant	3.9323***	3.9511***
	(0.1108)	(0.1112)
*Control*	Yes	Yes
*Industry FE*	Yes	Yes
*YearFE*	Yes	Yes
*N*	3086	3172
*R* ^*2*^	0.0865	0.0888

Note: One-to-four matching ensures that there is no substantial disparity in the means of the covariates, hence fulfilling the balancing assumption. For further details, please consult S1 Appendix. The report includes robust standard errors that are clustered at the firm level, indicated in brackets. ***p < 0.01; **p < 0.05; *p < 0.1.

#### 5.2.4. Omitted variable bias test.

In order to mitigate the potential bias caused by disregarded variables, we employ the approach developed by [87] to perform sensitivity studies on the extent of bias resulting from the omission of unseen factors in the measurement of observable variables. Our focus is specifically on two setting parameters: the selection ratio, denoted as δ, and the maximum fit of the regression equation, represented as $R_{max}$. In accordance with Oster’s (2019) proposal, we employ two methodologies to assess the empirical findings: (i) Set δ to 1 and let $R_{max}$ be 1.3 times the baseline regression $R^{\text{2}}$. The test is considered successful if the estimated parameter $\beta^{*}\left(R_{max},\delta \right)$ falls within the 95% confidence interval. (ii) Assume β is 0 and $R_{max}$ is 1.3 times the baseline regression $R^{\text{2}}$. If the calculated value of δ is less than 0, the test is considered successful. The outcomes of the test for omitted variable bias are displayed in Table 6. Column (2) displays the 95% confidence intervals for the variables SuppSt and Cust_stale in the benchmark regression. In column (3), we observe that the impact of SuppSt and Cust_stale on firms’ ESG performance, as measured by the parameter $\beta^{*}\left(R_{max},\delta \right)$ remains within the original 95% confidence ranges. Additionally, all of the values for δ are negative when β equals zero. Collectively, these findings indicate that our model configuration does not exhibit a significant issue of omitted variables.

**Table 6:** Omitted variable bias tests.

Variables	Standard	Estimated value	Omitted variables bias
SuppSt	$\beta^{*}\left(R_{max},\delta \right)\in [\text{0}.\text{0}\text{7}\text{4}\text{2},\text{0}.\text{4}\text{1}\text{6}\text{1}]$	$\beta^{*}\left(R_{max},\delta \right)=\text{0}.\text{2701}$	Unlikely
	δ<0	δ=−13.0308	Unlikely
Cust_stale	$\beta^{*}\left(R_{max},\delta \right)\in [\text{0}.\text{0}\text{4}\text{9}\text{1},\text{0}.\text{3}\text{1}\text{7}\text{6}]$	$\beta^{*}\left(R_{max},\delta \right)=\text{0}.\text{2072}$	Unlikely
	δ<0	δ=−11.1000	Unlikely

#### 5.2.5. Double machine learning.

Furthermore, we use the Double Machine Learning (DML) approach for endogeneity evaluation in order to solve the endogeneity problem and accurately estimate causality. Double Machine Learning (DML) is a state-of-the-art method that combines traditional econometric models with machine learning algorithms to improve causal inference in high-dimensional data analysis. It shows particular promise in resolving the endogeneity problem. In order to provide accurate treatment impact assessments, Dual Machine Learning (DML) essentially uses machine learning techniques to model and adjust for potentially endogenous influences. There are two separate stages to the DML approach. In order to solve the endogeneity problem between the dependent and independent variables, machine learning algorithms first forecast the explanatory, instrumental, and treatment factors by eliminating the components that are connected with other variables. Regression analysis is used in the second phase to evaluate the effects of therapy and policy. To find the best control variables, DML uses machine learning techniques like random forests and Lasso regression. These techniques are then integrated with traditional regression studies to ensure reliable and effective results. To address the endogeneity issues, following the step of Zhang (2023), we first developed instrumental variables for dual machine learning in this study. For endogeneity testing, we calculated industry averages for customer and supply chain stability by industry classification and developed instrumental variables by cubing the sample-to-mean difference. The explanatory, treatment, and instrumental variables were then predicted using Lasso regression, with cross-validation used to optimize the model and avoid overfitting. Finally, we obtained the modified regression coefficients, which showed that supply chain and customer stability had a considerable favorable impact on businesses’ ESG performance. Additionally, after adding the control variables’ quadratic components and running more tests, the regression coefficients continued to be statistically positive, as displayed in Table 7. The soundness of the econometric model was confirmed by its impartiality and consistency.

**Table 7:** Double Machine Learning.

	(1)	(2)	(3)	(4)
	ESG_grade	ESG_grade	ESG_grade	ESG_grade
SuppSt	0.2283***	0.2112***		
	(0.0788)	(0.0790)		
Cust_stale			0.2444***	0.2220***
			(0.0633)	(0.0630)
*Control variable a term*	Yes	Yes	Yes	Yes
*Control variable quadratic term*	No	Yes	No	Yes
*Industry FE*	Yes	Yes	Yes	Yes
*YearFE*	Yes	Yes	Yes	Yes
*N*	3530	3530	3530	3530

### 5.3. Robustness test

In this section, we perform a number of supplementary tests to verify the strength and reliability of our primary conclusions. In Table 8, we utilise the proportion of consecutive years in which the first major supplier/customer remains unchanged (SuppSth/ Cust_staleh) as a proxy variable to measure the stability of enterprises’ supply chains. More precisely, we categorise companies into high and low categories by calculating the median percentage of years in which the initial supplier or client remains unchanged. A corporation is deemed to possess great supplier/customer stability when the percentage of consecutive years without any alteration in its primary supplier/customer is above the median, which is designated as a value of 1, and 0 otherwise. We modify the assessment of enterprises’ ESG performance in columns (3) and (4). We employ a single proxy variable to assess the ESG performance of companies. To approximate the ESG performance of firms, we utilise the firm’s relevant CSI ESG composite score and divide it by 100, treating it as a proxy variable. Furthermore, the quantile regression model offers a more extensive range of information at the quantile level, in contrast to the OLS model. Additionally, it overcomes the limitations of the OLS model, which is vulnerable to extreme values. Hence, we employ quantile regression models in columns (5) and (6) to examine our primary findings. In Table 9, columns (1) to (6), we eliminate companies with unsatisfactory disclosure outcomes from the Shenzhen Stock Exchange (SSE), companies with objectives related to both maximum carbon emissions and carbon neutrality and companies situated in provinces and cities participating in carbon trading pilot programmes. This is due to the fact that the ESG performance of these organisations is more susceptible to the impact of pertinent laws and regulations. For each of these instances, the supply chain stability indicator shows a notably favourable result, with a statistical significance of at least 10 per cent. This indicates that our findings remain strong and reliable.

**Table 8:** Robustness test: Alternative model and metric.

	(1)	(2)	(3)	(4)	(5)	(6)
SuppSt			0.0133***		0.0106***	
			(0.0043)		(0.0034)	
Cust_stale				0.0090***		0.0084***
				(0.0034)		(0.0030)
SuppSth	0.0926*					
	(0.0512)					
Cust_staleh		0.0921*				
		(0.0478)				
Constant	4.0172***	4.0168***	0.7220***	0.7238***	0.7232***	0.7205***
	(0.1007)	(0.1011)	(0.0056)	(0.0055)	(0.0085)	(0.0114)
*Control*	Yes	Yes	Yes	Yes	Yes	Yes
*Industry FE*	Yes	Yes	Yes	Yes	Yes	Yes
*Year FE*	Yes	Yes	Yes	Yes	Yes	Yes
*N*	3530	3530	3530	3530	3530	3530
*Pseudo-R* ^*2*^ */R* ^*2*^	0.0888	0.0889	0.0971	0.0957	0.0561	0.0561

**Notes:** The dependent variable in columns (5) and (6) for the quantile regression model is the CSI ESG composite score divided by 100. The provided standard errors are robust and enclosed in brackets. The report includes robust standard errors that are clustered at the firm level, indicated in brackets. ***p < 0.01; **p < 0.05; *p < 0.1.

**Table 9:** Robustness test: Excluding special samples.

	(1)	(2)	(3)	(4)	(5)	(6)
SuppSt	0.2375***		0.2814***		0.1959*	
	(0.0868)		(0.0940)		(0.1022)	
Cust_stale		0.1612**		0.2155***		0.2312***
		(0.0689)		(0.0751)		(0.0822)
Constant	3.9650***	3.9974***	4.0332***	4.0578***	3.8006***	3.7744***
	(0.1075)	(0.1064)	(0.1138)	(0.1137)	(0.1464)	(0.1438)
*Control*	Yes	Yes	Yes	Yes	Yes	Yes
*Industry FE*	Yes	Yes	Yes	Yes	Yes	Yes
*Year FE*	Yes	Yes	Yes	Yes	Yes	Yes
*N*	3470	3470	2758	2758	2444	2444
*R* ^*2*^	0.0914	0.0902	0.1055	0.1046	0.1011	0.1029

Note: The report includes robust standard errors that are clustered at the firm level, indicated in brackets. ***p < 0.01; **p < 0.05; *p < 0.1.

### 5.4. Analysis of mechanisms

This section examines the manner in which the stability of the supply chain impacts the environmental, social, and governance (ESG) performance of corporations. This article examines the impact of supply chain stability on company ESG performance, focusing on business risk-taking, corporate agency costs, and corporate financing restrictions.

This study examines the impact of corporate risk-taking on supply chain stability and corporate ESG performance. To measure corporate risk-taking, the standard deviation of the three-year and five-year corporate net profit margin of total assets is used. The regression analysis between corporate supply chain stability, customer stability, and risk-taking is presented in Table 10. The regression coefficients in columns (1) to (4) are significantly negative, indicating that an increase in supply chain stability leads to a reduction in risk-taking. This is achieved by enhancing supplier stability and improving the enterprise’s ability to fulfill contracts. Furthermore, lower business risk allows the enterprise to allocate more resources to corporate social responsibility, leading to an improvement in the enterprise’s ESG rating. Therefore, H2 is supported. Furthermore, the magnitude of the regression coefficient for supplier stability is double that of customer stability, suggesting that supplier stability has a more significant influence on mitigating business risk.

**Table 10:** Mechanism: Corporate risk-taking.

	(1)	(2)	(3)	(4)
	Three_RiskTaking	Three_RiskTaking	Five_RiskTaking	Five_RiskTaking
SuppSt	−3.1959***		−3.9306***	
	(0.9467)		(1.0561)	
Cust_stale		−1.3499*		−1.8864**
		(0.7648)		(0.8876)
	(2.5190)	(2.5190)	(2.9802)	(2.9681)
Constant	7.3840***	6.6190***	9.7197***	8.8790***
	(0.9501)	(0.9521)	(1.1768)	(1.1938)
*Control*	Yes	Yes	Yes	Yes
*Industry FE*	Yes	Yes	Yes	Yes
*Year FE*	Yes	Yes	Yes	Yes
*N*	2758	2758	2758	2758
*R* ^*2*^	0.1054	0.1012	0.1061	0.1006

**Notes:** The report includes robust standard errors that are clustered at the firm level, indicated in brackets. ***p < 0.01; **p < 0.05; *p < 0.1.

This study examines the impact of agency cost on supply chain stability and its effect on enterprise ESG performance. The agency cost of enterprises is measured using the ratio of management expenses to operating income. The regression analysis, presented in Table 11, shows a significant negative relationship between supply chain stability and agency cost. This suggests that maintaining stable supply chain relationships can effectively reduce agency cost by minimizing internal opportunistic behaviors. External supervision within supply chain networks helps in achieving information sharing among enterprises. By externally supervising the relevant enterprises in the supply chain network, it is possible to decrease opportunistic behaviors within the enterprise, enhance operational transparency, foster corporate governance, and facilitate information sharing among the enterprises in the supply chain network. Therefore, H3 has been confirmed.

**Table 11:** Mechanism: Agency cost.

	(1)	(2)
	Agency cost	Agency cost
SuppSt	−0.0088***	
	(0.0020)	
Cust_stale		−0.0068***
		(0.0017)
Constant	0.0298***	0.0289***
	(0.0025)	(0.0024)
*Control*	Yes	Yes
*Industry FE*	Yes	Yes
*Year FE*	Yes	Yes
*N*	3513	3513
*R* ^*2*^	0.1449	0.1435

Note: The report includes robust standard errors that are clustered at the firm level, indicated in brackets. ***p < 0.01; **p < 0.05; *p < 0.1.

In order to test the role of financing constraints in the mechanism of supply chain stability to promote the enhancement of enterprise ESG performance, this paper selects the KZ coefficient to measure the financing constraints of enterprises, the regression results of the enterprise supply chain stability on the financing constraints of enterprises are shown in Table 11, the regression coefficients of the supply chain stability on the financing constraints are significantly negative at the significance level of 1 per cent, which indicates that the enterprises in the more stable supply chain network relying on the endorsement of the whole chain enterprises’ endorsement can have lower financing constraints, and this endorsement relies on the joint development of enterprises in the whole chain, and in the context of cash flow security, enterprises will also have the ability to optimise ESG performance, and the enterprises in the supply chain network together to optimise ESG performance, H4 is proved Table 12.

**Table 12:** Mechanism: Financing constraints.

	(1)	(2)
	Financing constraints	Financing constraints
SuppSt	−0.2518***	
	(0.0718)	
Cust_stale		−0.3138***
		(0.0798)
Constant	4.2991***	4.3283***
	(0.1312)	(0.1328)
*Control*	Yes	Yes
*Industry FE*	Yes	Yes
*Year FE*	Yes	Yes
*N*	3522	3522
*R* ^*2*^	0.6433	0.6459

Note: The report includes robust standard errors that are clustered at the firm level, indicated in brackets. ***p < 0.01; **p < 0.05; *p < 0.1.

### 5.5. Heterogeneity test

To examine the differential impact of supply chain stability on firms’ ESG performance, firms were categorised according to factors such as firm size, bank share ownership, audit quality, geographical location, executive traits and production intensity. This analysis highlights how these factors influence the relationship between supply chain stability and ESG performance.

In the grouped regression analysis of supplier stability, enterprises are first classified according to their scale and whether they hold bank stocks. The regression coefficient of large enterprises (i.e., those above the average size) is significantly positive, indicating that the stability of suppliers can significantly enhance their ESG performance. In contrast, the regression coefficients of small enterprises are not significant, indicating that the stability of suppliers has a relatively weak impact on their ESG performance. Further grouping difference tests revealed that the difference in the impact of supplier stability on ESG performance between large enterprises and small enterprises was significant at the 1% significance level. This might be attributed to the fact that large enterprises have stronger advantages in bargaining power, market position, and maintaining a stable supply chain, while small enterprises rely more on cost-effective business strategies. In terms of whether enterprises hold bank stocks, the regression coefficient of enterprises that do not hold bank stocks is significantly positive, while that of enterprises that hold bank stocks is not significant. Moreover, the difference between the two groups is significant at the 5% significance level. Enterprises holding bank stocks have advantages in capital operation and financing channels, and thus are relatively less dependent on stable supply chains. Their ESG performance improvement is less sensitive to the stability of suppliers. On the contrary, non-shareholding enterprises face stricter capital constraints and operational risks, and rely more on stable supplier relationships to ensure normal operation and promote long-term development. It can be seen that supplier stability has a more obvious positive impact on the ESG performance of large enterprises and non-shareholding enterprises, highlighting the interaction among enterprise scale, financial autonomy and supply chain management in influencing ESG performance Table 13.

**Table 13:** Heterogeneity test: Grouping based on supplier stability.

	(1)	(2)	(3)	(4)
	ESG_grade (Big size)	ESG_grade (Small size)	ESG_grade (Hold bank shares)	ESG_grade (No bank shares)
SuppSt	0.3961***	0.0803	−0.1142	0.2904***
	(0.1250)	(0.1077)	(0.2401)	(0.0914)
_cons	4.1263***	3.7646***	4.2205***	3.8789***
	(0.1739)	(0.1350)	(0.3407)	(0.1136)
	P = 0.01		P = 0.05	
Control	Yes	Yes	Yes	Yes
Industry FE	Yes	Yes	Yes	Yes
Year FE	Yes	Yes	Yes	Yes
N	1595	1933	234	3294
R^2^	0.1238	0.1438	0.3582	0.0932

Note: The report includes robust standard errors that are clustered at the firm level, indicated in brackets. ***p < 0.01; **p < 0.05; *p < 0.1.

Group regression analysis based on audit types and audit opinions shows that the coefficients of enterprises audited by the Big Four accounting firms are significantly positive, while the impact on non-Big Four audited enterprises is relatively small and less significant. This indicates that high-quality audits can enhance the positive effect of supplier stability on ESG performance. Similarly, among enterprises that received standard audit opinions, the impact of supplier stability on ESG performance was more significant, while for those that did not receive standard audit opinions, the impact was not significant. The inter-group difference test was significant at both 1% and 5% significance levels. This result indicates that enterprises with strict auditing and sound governance structures typically possess higher financial transparency, stronger compliance, and more effective risk management capabilities, and thus are better able to translate supplier stability into ESG performance improvements. In contrast, enterprises with lower audit quality or weak governance structures have limited response capabilities to external supply chain dynamics due to financial constraints and insufficient governance, and thus find it difficult to utilize supply chain stability to improve ESG performance. Overall, the research results highlight the moderating role of audit quality and governance level in enhancing the relationship between supplier stability and ESG performance Table 14.

**Table 14:** Heterogeneity test: Grouping based on supplier stability.

	(1)	(2)	(3)	(4)
	ESG_grade (With Big4)	ESG_grade (No Big4)	ESG_grade (Standard Opinion)	ESG_grade (No standard opinion)
SuppSt	1.2621***	0.2267**	0.2488***	−0.5121
	(0.4398)	(0.0884)	(0.0871)	(0.3806)
_cons	4.1383***	3.9451***	3.9408***	3.4973***
	(0.7712)	(0.1106)	(0.1121)	(0.4192)
	P = 0.01		P = 0.04	
Control	Yes	Yes	Yes	Yes
Industry FE	Yes	Yes	Yes	Yes
Year FE	Yes	Yes	Yes	Yes
N	99	3422	3384	138
R^2^	0.5223	0.0890	0.0927	0.4694

Note: The report includes robust standard errors that are clustered at the firm level, indicated in brackets. ***p < 0.01; **p < 0.05; *p < 0.1.

The grouped regression analysis based on the geographical location of enterprises shows that at the 1% significance level, the regression coefficient of enterprises in the western region is significantly positive, while the influence of enterprises in non-western regions is smaller and the significance is weaker. The differences between groups were equally significant at the 1% level. This result can be attributed to the special characteristics of enterprises in the western region. In the western regions, due to relatively low market competition and limited economic resources, enterprises rely more on stable customer relationships. Therefore, customer stability plays a more significant role in enhancing their ESG performance. In contrast, enterprises in non-western regions typically have more diversified businesses and more stable customer networks, and are relatively less dependent on customer stability for ESG performance.

Further grouped regression based on the listing locations of enterprises shows that the regression coefficient of listed enterprises on the Shenzhen Stock Exchange (SZSE) is significantly positive, while the coefficient of non-SZSE listed enterprises is not significant. The difference between the groups is significant at the 1% significance level. This result may be closely related to the policy-oriented characteristics of SZSE. SZSE has always attached great importance to innovation-driven development and supported small and medium-sized enterprises, especially technology-oriented and growth-oriented ones. Therefore, SZSE listed companies usually possess stronger innovation capabilities and higher market transparency, and pay more attention to customer stability to maintain long-term competitiveness and market position. On the contrary, non-SZSE listed companies may lack policy guidance and support, facing problems of lower market attention and resource allocation. As a result, they pay less attention to customer stability, thereby limiting their impact on ESG performance Table 15.

**Table 15:** Heterogeneity test: Grouping based on customer stability.

	(1)	(2)	(3)	(4)
	ESG_grade (West)	ESG_grade (Not West)	ESG_grade (Listed in Shenzhen)	ESG_grade (Not listed in Shenzhen)
Cust_stale	0.4616***	0.1281*	0.2377***	−0.1975
	(0.1590)	(0.0726)	(0.0739)	(0.1694)
_cons	2.9925***	4.0860***	3.8740***	4.4160***
	(0.3285)	(0.1107)	(0.1163)	(0.2608)
	P = 0.01		P = 0.000	
Control	Yes	Yes	Yes	Yes
Industry FE	Yes	Yes	Yes	Yes
Year FE	Yes	Yes	Yes	Yes
N	613	2912	2989	537
R^2^	0.3022	0.0963	0.1031	0.2231

Note: The report includes robust standard errors that are clustered at the firm level, indicated in brackets. ***p < 0.01; **p < 0.05; *p < 0.1.

The results of the grouped regression analysis based on the characteristics of senior executives show that the regression coefficient of the enterprises where ceos with financial backgrounds are located is moderately positive, while the significance level of the enterprises where ceos without financial backgrounds are relatively low. The differences between groups were significant at the 1% significance level. This indicates that ceos with financial experience typically possess stronger financial literacy and risk management capabilities, enabling them to maintain and utilize supply chain stability more effectively, thereby enhancing the ESG performance of the enterprise. This type of CEO is more inclined to focus on long-term strategy and financial sustainability, and can identify the crucial role of supplier and customer stability in enhancing corporate governance and social responsibility. In contrast, ceos without a financial background may lack experience in financial management and risk control. Even if they have professional capabilities in other management areas, this may lead to them paying less attention to supply chain stability and ESG performance.

Similarly, in the regression analysis grouped by technology intensity, the results showed that the regression coefficients of non-technology-intensive enterprises were moderately positive, while those of technology-intensive enterprises were not significant. The differences between groups were significant at the 1% significance level. This indicates that enterprises with lower technology intensity typically rely more on stable supply chain partnerships to ensure production continuity, thereby enhancing their ESG performance. For these enterprises, supply chain stability is the main means to achieve risk management and maintain competitive advantages. In contrast, technology-intensive enterprises typically require significant investment in research and development and are highly dependent on technological innovation. Their production and operation processes rely more on technological capabilities rather than traditional supply chain stability. Therefore, the impact of supply chain stability on their ESG performance is relatively small. Among these enterprises, the role of technological innovation in shaping market competitiveness and sustainable development may outweigh that of supply chain stability Table 16.

**Table 16:** Heterogeneity test: Grouping based on customer stability.

	(1)	(2)	(3)	(4)
	ESG_grade (CEOCW)	ESG_grade (No CEOCW)	ESG_grade (Technology-intensive)	ESG_grade (Non-technology-intensive)
Cust_stale	0.6004***	0.1491**	0.0487	0.2681***
	(0.2205)	(0.0744)	(0.1191)	(0.0840)
_cons	3.9053***	3.9728***	3.8828***	3.9356***
	(0.2612)	(0.1185)	(0.1751)	(0.1281)
	P = 0.01		P = 0.01	
Control	Yes	Yes	Yes	Yes
Industry FE	Yes	Yes	Yes	Yes
Year FE	Yes	Yes	Yes	Yes
N	383	2927	1280	2250
R^2^	0.3283	0.0918	0.1054	0.1001

Note: The report includes robust standard errors that are clustered at the firm level, indicated in brackets. ***p < 0.01; **p < 0.05; *p < 0.1.

## 6. Case study

As a globally leading lithium battery manufacturer, CATL (Contemporary Amperex Technology Co. Limited) is a typical case of supply chain resilience and ESG performance improvement. The company has significantly enhanced its ESG performance in supply chain management through green procurement, continuous cooperation and comprehensive risk management. According to CATL’s 2022 annual report, over 70% of its suppliers have been incorporated into its green supply chain system, reducing resource consumption by 30% and cutting annual pollutant emissions by more than 100 million tons. The company uses environmentally sustainable materials in its production process, promotes battery recycling, and employs renewable energy in approximately 90% of its manufacturing processes. Through these green supply chain measures, CATL has significantly reduced resource waste and carbon emissions in the raw material procurement process, thereby enhancing its ESG rating in the environmental protection dimension. In addition to environmental performance, CATL has also effectively reduced enterprise risks through a reliable supply chain. The company has established long-term cooperation agreements with the five major lithium ore suppliers, effectively mitigating the impact of raw material price fluctuations on production costs. Among them, the fluctuation range of lithium ore prices dropped by 35%, and the fluctuation of production costs narrowed from ±20% to ±8%. Through a diversified supplier network, the company has effectively avoided the risk of a single source, ensuring production continuity and market stability. Catl has also achieved remarkable results in reducing agency costs. The company has established strict environmental protection standards, signed transparent agreements with suppliers, and conducted regular supplier evaluations. This not only reduces information asymmetry in the supply chain but also enhances the collaborative relationship between the enterprise and its suppliers. As a result, the company’s operating costs dropped significantly in 2022, and the audit fees for supply chain management decreased by approximately 40% compared to 2020. In addition, the stability of the supply chain has significantly enhanced the company’s financing capacity. Catl’s solid ESG performance and commitment to sustainable development have enhanced its reputation in the capital market, thereby reducing its financing costs. For instance, in 2021, the company issued 5 billion yuan worth of green bonds with an interest rate of 3.5%, which was 0.7 percentage points lower than the 4.2% of enterprises in the same industry. Solid ESG performance and reliable supply chains have prompted financial institutions to offer low-interest financing, thereby supporting the expansion of green projects and technological innovation.This case demonstrates that supply chain stability is crucial for enhancing overall organization performance and achieving sustainable development, thereby offering valuable insights for companies seeking to improve ESG performance in supply chain management.

## 7. Conclusion

The severe risk of climate change makes the public pay more attention to the ESG performance of enterprises, and the uncertainty of the international political economy also focuses the attention of the society on the issue of supply chain breakage, and the research explores the relationship and influence mechanism between supply chain stability and enterprise ESG performance, which is of great practical significance and theoretical value for the development of the society and economy. This paper uses the data of Chinese A-share listed companies from 2009 to 2022 to examine the impact of supply chain stability on corporate ESG performance and explore the mechanism of the impact, confirming that the improvement of supply chain stability can improve corporate ESG performance, and the improvement of supply chain stability can optimise corporate ESG performance by reducing corporate risk-taking, agency costs, and financing constraints, etc. The promotion effect can be seen more significant in large firms, firms with higher-standard audit supervision, firms located in western regions, and non-technology-intensive firms.

Based on this, this study not only reveals the correlation between supply chain stability and the sustainable development of enterprises at the empirical level but also makes multiple contributions at both the theoretical and practical levels. From a theoretical perspective, first, this study has cross-integrated the two relatively independent research fields of supply chain management and enterprise sustainable development, expanding the existing ESG literature’s research perspective mainly focusing on internal governance or financial characteristics, and emphasizing the external supply chain relationship as an important exogenous governance mechanism influencing the sustainable behavior of enterprises. Secondly, this study integrates the resource dependence theory, social capital theory and agency theory into the same analytical framework, systematically explaining how supply chain stability improves the ESG performance of enterprises through paths such as resource allocation, governance efficiency and information transparency, enriching the theoretical explanation of the impact of supply chain governance on the non-financial performance of enterprises. Thirdly, this study reveals that supply chain stability shows heterogeneous effects in different enterprise types and institutional environments, which is conducive to deepening the understanding of the boundaries of the role of external governance mechanisms in different organizational contexts. From a practical perspective, the research results provide targeted inspirations for policymakers and business managers. First, local governments can promote the stable development of supply chains through financial and institutional support. By providing low-interest financing or green supply chain loans to large enterprises and core suppliers, they are encouraged to establish long-term cooperative relationships with upstream and downstream partners to ensure the flow of resources and the stability of funds. At the same time, a supply chain risk early warning system can be established to conduct dynamic supervision over key links and high-risk enterprises, thereby reducing the risk of supply chain disruption from the source. Secondly, the government and regulatory authorities should strengthen supervision and incentives over enterprises’ ESG practices. They should guide enterprises to actively engage in environmental and social responsibility construction through strict auditing systems, transparent information disclosure requirements, and tax preferential policies. Finally, enterprises in the upstream and downstream of the supply chain should collaborate to establish green and sustainable production management standards, set up an ESG assessment system for supply chain partners, and provide incentives to enterprises that perform outstandingly in environmental governance and social responsibility. This will create a virtuous cycle within the supply chain and enhance the overall sustainability level of the supply chain.

However, this article also has limitations, as it concentrates on Chinese A-share listed companies and does not account for the corporate behavior of unlisted companies and multinationals inside the global supply chain system. This study also neglects the influence of advancements in technology and the international social environment on corporate ESG development, including the effects of digital technology and artificial intelligence, as well as the connection between supply chain stability and ESG amid U.S.-China trade tensions and geopolitical conflicts. Future research may expand the sample to include multinational enterprises and unlisted companies, further examining the influence of technological advancements on corporate ESG strategies, as well as investigating the effects of supply chain disruptions on corporate ESG behaviors amid the uncertainties of global economic policies. Moreover, the enduring effects of enhanced corporate supply chain stability on corporate sustainability can be further progressed, and a more profound comprehension of how supply chain management optimization influences ESG performance over time can be cultivated.

## Supporting information

S1 File Opendata. (XLSX)

S1 Appendix Balance test. (DOCX)

## References

[1] McCulloch MT, Winter A, Sherman CE, Trotter JA. 300 years of sclerosponge thermometry shows global warming has exceeded 1.5 °C. Nat Clim Chang. 2024;14(2):171–7. doi: 10.1038/s41558-023-01919-7

[2] Matsumura EM, Prakash R, Vera-Muñoz SC. Climate-risk materiality and firm risk. Rev Account Stud. 2022;29(1):33–74. doi: 10.1007/s11142-022-09718-9

[3] Woetzel J, Pinner D, Samandari H. Climate risk and response: Physical hazards and socioeconomic impacts. 2020.

[4] Hübel B, Scholz H. Integrating sustainability risks in asset management: the role of ESG exposures and ESG ratings. J Asset Manag. 2019;21(1):52–69. doi: 10.1057/s41260-019-00139-z

[5] Mooneeapen O, Abhayawansa S, Mamode Khan N. The influence of the country governance environment on corporate environmental, social and governance (ESG) performance. SAMPJ. 2022;13(4):953–85. doi: 10.1108/sampj-07-2021-0298

[6] Kim S, Yoon A. Analyzing Active Fund Managers’ Commitment to ESG: Evidence from the United Nations Principles for Responsible Investment. Management Science. 2023;69(2):741–58. doi: 10.1287/mnsc.2022.4394

[7] Dai T, Tang C. Frontiers in service science: Integrating ESG Measures and Supply Chain Management: Research Opportunities in the Postpandemic Era. Service Science. 2022;14(1):1–12. doi: 10.1287/serv.2021.0295

[8] Vertinsky I, Kuang Y, Zhou D, Cui V. The political economy and dynamics of bifurcated world governance and the decoupling of value chains: An alternative perspective. J Int Bus Stud. 2023;:1–27. doi: 10.1057/s41267-023-00597-z 36743261 PMC9886532

[9] Tsang YP, Fan Y, Feng ZP, Li Y. Examining supply chain vulnerability via an analysis of ESG-Prioritized firms amid the Russian-Ukrainian conflict. Journal of Cleaner Production. 2024;434:139754. doi: 10.1016/j.jclepro.2023.139754

[10] Al-Omoush KS, de Lucas A, del Val MT. The role of e-supply chain collaboration in collaborative innovation and value-co creation. Journal of Business Research. 2023;158:113647. doi: 10.1016/j.jbusres.2023.113647

[11] Tyteca D. On the measurement of the environmental performance of firms— A literature review and a productive efficiency perspective. Journal of Environmental Management. 1996;46(3):281–308. doi: 10.1006/jema.1996.0022

[12] Zhen X, Shi D, Li Y, Zhang C. Manufacturer’s financing strategy in a dual-channel supply chain: Third-party platform, bank, and retailer credit financing. Transportation Research Part E: Logistics and Transportation Review. 2020;133:101820. doi: 10.1016/j.tre.2019.101820

[13] Kamalahmadi M, Shekarian M, Mellat Parast M. The impact of flexibility and redundancy on improving supply chain resilience to disruptions. International Journal of Production Research. 2021;60(6):1992–2020. doi: 10.1080/00207543.2021.1883759

[14] Vachon S, Klassen RD. Extending green practices across the supply chain. International Journal of Operations & Production Management. 2006;26(7):795–821. doi: 10.1108/01443570610672248

[15] Zhang J, Mo H, Hu Z, Zhang T. The effect of stability and concentration of upstream and downstream relationships of focal firms on two-level trade credit. International Journal of Production Economics. 2024;270:109173. doi: 10.1016/j.ijpe.2024.109173

[16] Zi Y. Trade costs, global value chains and economic development. Journal of Economic Geography. 2020;20:249–91.

[17] Levy H, Sarnat M. International diversification of investment portfolios. The American Economic Review. 1970;60:668–75.

[18] McMaster M, Nettleton C, Tom C, Xu B, Cao C, Qiao P. Risk management: Rethinking Fashion Supply Chain Management for Multinational Corporations in Light of the COVID-19 Outbreak. JRFM. 2020;13(8):173. doi: 10.3390/jrfm13080173

[19] Aydoğmuş M, Gülay G, Ergun K. Impact of ESG performance on firm value and profitability. Borsa Istanbul Review. 2022;22:S119–27. doi: 10.1016/j.bir.2022.11.006

[20] Geng X, Han B, Yang D, Zhao J. Credit risk contagion of supply chain finance: An empirical analysis of supply chain listed companies. PLoS One. 2024;19(8):e0306724. doi: 10.1371/journal.pone.0306724 39190762 PMC11349223

[21] Li X, Wu Q, Holsapple CW, Goldsby T. An empirical examination of firm financial performance along dimensions of supply chain resilience. MRR. 2017;40(3):254–69. doi: 10.1108/mrr-02-2016-0030

[22] Ahmed MU, Shafiq A. Toward sustainable supply chains: impact of buyer’s legitimacy, power and aligned focus on supplier sustainability performance. IJOPM. 2022;42(3):280–303. doi: 10.1108/ijopm-08-2021-0540

[23] Yao R, Fei Y, Wang Z, Yao X, Yang S. The Impact of China’s ETS on corporate green governance based on the perspective of corporate ESG performance. Int J Environ Res Public Health. 2023;20(3):2292. doi: 10.3390/ijerph20032292 36767659 PMC9915039

[24] Bisetti E, She G, Zaldokas A. ESG shocks in global supply chains. 2023.

[25] Makris D, Hansen ZNL, Khan O. Adapting to supply chain 4.0: An explorative study of multinational companies. Supply Chain Forum: An International Journal. 2019;20(2):116–31. doi: 10.1080/16258312.2019.1577114

[26] Hugos MH. Essentials of supply chain management. John Wiley & Sons. 2024.

[27] Nayal K, Raut RD, Yadav VS, Priyadarshinee P, Narkhede BE. The impact of sustainable development strategy on sustainable supply chain firm performance in the digital transformation era. Bus Strat Env. 2024;33(5):4974–5. doi: 10.1002/bse.3783

[28] Wieland A, Durach CF. Two perspectives on supply chain resilience. Wiley Online Library. 2021.

[29] Lai K, Cheng TCE, Yeung ACL. Relationship stability and supplier commitment to quality. International Journal of Production Economics. 2005;96(3):397–410. doi: 10.1016/j.ijpe.2004.07.005

[30] Wiegel W, Bamford D. The role ofguanxiin buyer–supplier relationships in Chinese small- and medium-sized enterprises – A resource-based perspective. Production Planning & Control. 2014;:1–20. doi: 10.1080/09537287.2014.899405

[31] Pant P, Dutta S, Sarmah SP. Supply chain relational capital and firm performance: an empirical enquiry from India. IJOEM. 2022;19(1):76–105. doi: 10.1108/ijoem-05-2021-0663

[32] Cannon JP, Homburg C. Buyer–supplier relationships and customer firm costs. Journal of Marketing. 2001;65(1):29–43. doi: 10.1509/jmkg.65.1.29.18136

[33] Balke N, Lamadon T. Productivity shocks, long-term contracts, and earnings dynamics. American Economic Review. 2022;112(7):2139–77. doi: 10.1257/aer.20161622

[34] Johnsen RE, Lacoste S, Meehan J. Hegemony in asymmetric customer-supplier relationships. Industrial Marketing Management. 2020;87:63–75. doi: 10.1016/j.indmarman.2020.01.013

[35] Korpeoglu CG, Körpeoğlu E, Cho S-H. Supply chain competition: A market game approach. Management Science. 2020;66(12):5648–64. doi: 10.1287/mnsc.2019.3511

[36] Wang Y, Ha AY, Tong S. Sharing Manufacturer’s demand information in a supply chain with price and service effort competition. M&SOM. 2022;24(3):1698–713. doi: 10.1287/msom.2021.1028

[37] Ostrovsky M. Stability in supply chain networks. American Economic Review. 2008;98(3):897–923. doi: 10.1257/aer.98.3.897

[38] Sciascia I. Customer Loyalty as Measure of Competitiveness. IJMS. 2022;14(1):1. doi: 10.5539/ijms.v14n1p1

[39] Chatterjee L, Feng C, Nakata C, Sivakumar K. The environmental turbulence concept in marketing: A look back and a look ahead. Journal of Business Research. 2023;161:113775. doi: 10.1016/j.jbusres.2023.113775

[40] Rane NL, Achari A, Choudhary SP. Enhancing customer loyalty through quality of service: Effective strategies to improve customer satisfaction, experience, relationship, and engagement. International Research Journal of Modernization in Engineering Technology and Science. 2023;5:427–52.

[41] Guerola-Navarro V, Gil-Gomez H, Oltra-Badenes R, Sendra-García J. Customer relationship management and its impact on innovation: A literature review. Journal of Business Research. 2021;129:83–7. doi: 10.1016/j.jbusres.2021.02.050

[42] Chatterjee S, Chaudhuri R, Vrontis D, Thrassou A, Ghosh SK, Chaudhuri S. Social customer relationship management factors and business benefits. IJOA. 2020;29(1):35–58. doi: 10.1108/ijoa-11-2019-1933

[43] Krueger P, Sautner Z, Tang DY, Zhong R. The effects of mandatory ESG disclosure around the world. J of Accounting Research. 2024;62(5):1795–847. doi: 10.1111/1475-679x.12548

[44] Freedman M, Jaggi B. Global warming, commitment to the Kyoto protocol, and accounting disclosures by the largest global public firms from polluting industries. The International Journal of Accounting. 2005;40(3):215–32. doi: 10.1016/j.intacc.2005.06.004

[45] Alrazi B, de Villiers C, Van Staden CJ. The environmental disclosures of the electricity generation industry: A global perspective. Accounting and Business Research. 2016;46(6):665–701. doi: 10.1080/00014788.2015.1135781

[46] de Aguiar TRS, Bebbington J. Disclosure on climate change: Analysing the UK ETS effects. Accounting Forum. 2014;38(4):227–40. doi: 10.1016/j.accfor.2014.10.002

[47] Luo L, Tang Q. Determinants of the quality of corporate carbon management systems: An international study. The International Journal of Accounting. 2016;51(2):275–305. doi: 10.1016/j.intacc.2016.04.007

[48] Avramov D, Cheng S, Lioui A, Tarelli A. Sustainable investing with ESG rating uncertainty. Journal of Financial Economics. 2022;145(2):642–64. doi: 10.1016/j.jfineco.2021.09.009

[49] Liu X, Cifuentes-Faura J, Zhao S, Wang L. The impact of government environmental attention on firms’ ESG performance: Evidence from China. Research in International Business and Finance. 2024;67:102124. doi: 10.1016/j.ribaf.2023.102124

[50] de Freitas Netto SV, Sobral MFF, Ribeiro ARB, Soares GR da L. Concepts and forms of greenwashing: A systematic review. Environ Sci Eur. 2020;32(1). doi: 10.1186/s12302-020-0300-3

[51] Ott C, Schiemann F, Günther T. Disentangling the determinants of the response and the publication decisions: The case of the Carbon Disclosure Project. Journal of Accounting and Public Policy. 2017;36(1):14–33. doi: 10.1016/j.jaccpubpol.2016.11.003

[52] Dupire M, Filbien J-Y, M’Zali B. Non-Governmental Organization (NGO) Tweets: Do Shareholders Care?. Business & Society. 2021;61(2):419–56. doi: 10.1177/0007650320985204

[53] Wong JB, Zhang Q. Stock market reactions to adverse ESG disclosure via media channels. The British Accounting Review. 2022;54(1):101045. doi: 10.1016/j.bar.2021.101045

[54] Park SR, Jang JY. The Impact of ESG Management on Investment Decision: Institutional Investors’ Perceptions of Country-Specific ESG Criteria. IJFS. 2021;9(3):48. doi: 10.3390/ijfs9030048

[55] Raimo N, Vitolla F, Marrone A, Rubino M. The role of ownership structure in integrated reporting policies. Bus Strat Env. 2020;29(6):2238–50. doi: 10.1002/bse.2498

[56] Morris M. Chinese firms and adherence to global Environmental, Social and Governance (ESG) standards in developing countries: Is there potential to create common ground?. 2023.

[57] Signori S, San-Jose L, Retolaza JL, Rusconi G. Stakeholder value creation: Comparing ESG and value added in European Companies. Sustainability. 2021;13(3):1392. doi: 10.3390/su13031392

[58] Aureli S, Del Baldo M, Lombardi R, Nappo F. Nonfinancial reporting regulation and challenges in sustainability disclosure and corporate governance practices. Bus Strat Env. 2020;29(6):2392–403. doi: 10.1002/bse.2509

[59] Zhang H, Tai H. The ESG disclosure and the stock price crash risk-evidence from China. Handelshøyskolen BI. 2023.

[60] Aghamolla C, An BJ. Mandatory vs. voluntary ESG disclosure, efficiency, and real effects. Nanyang Business School Research Paper, 2023.

[61] Nahapiet J, Ghoshal S. Social capital, intellectual capital, and the organizational advantage. Academy of Management Review. 1998;23(2):242–66.

[62] Pfeffer J, Salancik G. External control of organizations—Resource dependence perspective. Organizational behavior 2. Routledge. 2015. 355–70.

[63] Hillman AJ, Withers MC, Collins BJ. Resource Dependence Theory: A Review. Journal of Management. 2009;35(6):1404–27. doi: 10.1177/0149206309343469

[64] Ulrich D, Barney JB. Perspectives in Organizations: Resource dependence, efficiency, and population. AMR. 1984;9(3):471–81. doi: 10.5465/amr.1984.4279680

[65] Dyer JH, Singh H. The relational view: Cooperative strategy and sources of interorganizational competitive advantage. The Academy of Management Review. 1998;23(4):660. doi: 10.2307/259056

[66] Coleman JS. Social capital in the creation of human capital. American Journal of Sociology. 1988;94:S95–120. doi: 10.1086/228943

[67] Putnam RD. The prosperous community. The American Prospect. 1993;4(13):35–42.

[68] Adler PS, Kwon S-W. Social capital: Prospects for a new concept. The Academy of Management Review. 2002;27(1):17. doi: 10.2307/4134367

[69] Jones C, Hesterly WS, Borgatti SP. A general theory of network governance: Exchange conditions and social mechanisms. The Academy of Management Review. 1997;22(4):911. doi: 10.2307/259249

[70] Shi M, Yu W. Supply chain management and financial performance: Literature review and future directions. International Journal of Operations & Production Management. 2013;33(10):1283–317. doi: 10.1108/ijopm-03-2012-0112

[71] Wagner SM, Bode C. An empirical examination of supply chain performance along several dimensions of risk. J of Business Logistics. 2008;29(1):307–25. doi: 10.1002/j.2158-1592.2008.tb00081.x

[72] Christopher M, Peck H. Building the resilient supply chain. 2004.

[73] Manfredi E, Capik P. A case of trust‐building in the supply chain: Emerging economies perspective. Strategic Change. 2022;31(1):147–60. doi: 10.1002/jsc.2488

[74] Chowdhury MMH, Islam MT, Ali I, Quaddus M. The role of social capital, resilience, and network complexity in attaining supply chain sustainability. Bus Strat Env. 2023;33(3):2621–39. doi: 10.1002/bse.3613

[75] Zhu Q, Sarkis J, Lai K. Green supply chain management innovation diffusion and its relationship to organizational improvement: An ecological modernization perspective. Journal of Engineering and Technology Management. 2012;29(1):168–85. doi: 10.1016/j.jengtecman.2011.09.012

[76] Gimenez C, Sierra V. Sustainable supply chains: Governance mechanisms to greening suppliers. J Bus Ethics. 2012;116(1):189–203. doi: 10.1007/s10551-012-1458-4

[77] Kovács G. Corporate environmental responsibility in the supply chain. Journal of Cleaner Production. 2008;16(15):1571–8. doi: 10.1016/j.jclepro.2008.04.013

[78] Jensen MC, Meckling WH. Theory of the firm: Managerial behavior, agency costs and ownership structure. Corporate governance. Gower. 2019. 77–132.

[79] Cao M, Zhang Q. Supply chain collaboration: Impact on collaborative advantage and firm performance. J of Ops Management. 2010;29(3):163–80. doi: 10.1016/j.jom.2010.12.008

[80] Hoberg G, Phillips G, Prabhala N. Product market threats, payouts, and financial flexibility. The Journal of Finance. 2014;69(1):293–324. doi: 10.1111/jofi.12050

[81] Garcia AS, Mendes-Da-Silva W, Orsato RJ. Sensitive industries produce better ESG performance: Evidence from emerging markets. Journal of Cleaner Production. 2017;150:135–47. doi: 10.1016/j.jclepro.2017.02.180

[82] Cen L, Dasgupta S, Elkamhi R, Pungaliya RS. Reputation and Loan Contract Terms: The Role of Principal Customers. Review of Finance. 2015;20(2):501–33. doi: 10.1093/rof/rfv014

[83] Upson JE, Wei C. Supply chain concentration and cost of capital. Accounting & Finance. 2023;64(1):607–34. doi: 10.1111/acfi.13156

[84] Myers SC, Majluf NS. Corporate financing and investment decisions when firms have information that investors do not have. Journal of Financial Economics. 1984;13(2):187–221. doi: 10.1016/0304-405x(84)90023-0

[85] El Ghoul S, Guedhami O, Kim H, Park K. Corporate environmental responsibility and the cost of capital: International evidence. J Bus Ethics. 2016;149(2):335–61. doi: 10.1007/s10551-015-3005-6

[86] Lewbel A. Constructing instruments for regressions with measurement error when no additional data are available, with an application to patents and R&D. Econometrica. 1997;65(4):1201–13.

[87] Ortas E, M. Moneva J, Álvarez I. Sustainable supply chain and company performance. Supply Chain Management: An International Journal. 2014;19(3):332–50. doi: 10.1108/scm-12-2013-0444

