# Supplementary material for: A full belly counsels well: More stabilisation with more responsibility
Source: PLoS One. 2026 Mar 6;21(3):e0344283. doi: 10.1371/journal.pone.0344283 (PMC12965613; doi:10.1371/journal.pone.0344283)
Supplement: S1 Appendix — (DOCX) [file pone.0344283.s002.docx]

**S1** **Appendix. Balance test**

| Panel A: Balance test (Supplier) | | | | | | |
| --- | --- | --- | --- | --- | --- | --- |
| Variable | Unmatched and Matched | Treated group mean | Control group mean | %bias | t-value | P-value |
| REC | Unmatched | 0.1063 | 0.1183 | -11.5 | -3.33 | 0.001 |
|  | Matched | 0.1062 | 0.1047 | 1.5 | 0.43 | 0.671 |
| INV | Unmatched | 0.1218 | 0.1311 | -7.3 | -2.11 | 0.035 |
|  | Matched | 0.1219 | 0.1224 | -0.4 | -0.12 | 0.904 |
| Quick | Unmatched | 1.8214 | 1.8927 | -1.9 | -0.54 | 0.587 |
|  | Matched | 1.8248 | 1.8030 | 0.6 | 0.15 | 0.880 |
| ATO | Unmatched | 0.6708 | 0.6277 | 6.3 | 1.80 | 0.072 |
|  | Matched | 0.6698 | 0.6993 | -4.3 | -0.96 | 0.335 |
| FIXED | Unmatched | 0.2549 | 0.2218 | 18.8 | 5.48 | 0.000 |
|  | Matched | 0.2550 | 0.2557 | -0.4 | -0.10 | 0.916 |
| Panel B: Balance test (Customer) | | | | | | |
| Variable | Unmatched and Matched | Treated group mean | Control group mean | %bias | t-value | P-value |
| REC | Unmatched | 0.1061 | 0.1216 | -14.7 | -4.37 | 0.000 |
|  | Matched | 0.1064 | 0.1061 | 0.3 | 0.09 | 0.927 |
| INV | Unmatched | 0.1135 | 0.1430 | -22.9 | -6.84 | 0.000 |
|  | Matched | 0.1135 | 0.1153 | -1.4 | -0.49 | 0.622 |
| Quick | Unmatched | 1.8948 | 1.8293 | 1.7 | 0.51 | 0.613 |
|  | Matched | 1.8259 | 1.9615 | -3.6 | -0.97 | 0.331 |
| ATO | Unmatched | 0.6554 | 0.6344 | 3 | 0.89 | 0.374 |
|  | Matched | 0.6547 | 0.6598 | -0.7 | -0.22 | 0.825 |
| FIXED | Unmatched | 0.2636 | 0.2044 | 34 | 10.06 | 0.000 |
|  | Matched | 0.2630 | 0.2611 | 1.1 | 0.31 | 0.754 |
